# Unique Shine–Dalgarno Sequences in Cyanobacteria and Chloroplasts Reveal Evolutionary Differences in Their Translation Initiation

**DOI:** 10.1093/gbe/evz227

**Published:** 2019-10-22

**Authors:** Yulong Wei, Xuhua Xia

**Affiliations:** 1 Department of Biology, University of Ottawa, Ontario, Canada; 2 Ottawa Institute of Systems Biology, University of Ottawa, Ontario, Canada

**Keywords:** Cyanobacteria, chloroplast, RNA-Seq, bacterial translation initiation, Shine–Dalgarno, 16S rRNA

## Abstract

Microorganisms require efficient translation to grow and replicate rapidly, and translation is often rate-limited by initiation. A prominent feature that facilitates translation initiation in bacteria is the Shine–Dalgarno (SD) sequence. However, there is much debate over its conservation in Cyanobacteria and in chloroplasts which presumably originated from endosymbiosis of ancient Cyanobacteria. Elucidating the utilization of SD sequences in Cyanobacteria and in chloroplasts is therefore important to understand whether 1) SD role in Cyanobacterial translation has been reduced prior to chloroplast endosymbiosis or 2) translation in Cyanobacteria and in plastid has been subjected to different evolutionary pressures. To test these alternatives, we employed genomic, proteomic, and transcriptomic data to trace differences in SD usage among *Synechocystis* species, *Microcystis aeruginosa*, cyanophages, *Nicotiana tabacum* chloroplast, and *Arabidopsis thaliana* chloroplast. We corrected their mis-annotated 16S rRNA 3′ terminus using an RNA-Seq-based approach to determine their SD/anti-SD locational constraints using an improved measurement *D*_toStart_. We found that cyanophages well-mimic Cyanobacteria in SD usage because both have been under the same selection pressure for SD-mediated initiation. Whereas chloroplasts lost this similarity because the need for SD-facilitated initiation has been reduced in plastids having much reduced genome size and different ribosomal proteins as a result of host-symbiont coevolution. Consequently, SD sequence significantly increases protein expression in Cyanobacteria but not in chloroplasts, and only Cyanobacterial genes compensate for a lack of SD sequence by having weaker secondary structures at the 5′ UTR. Our results suggest different evolutionary pressures operate on translation initiation in Cyanobacteria and in chloroplast.

## Introduction

In bacteria, initiation is often the rate limiting step in translation (Andersson and Kurland 1983; Kudla et al. 2009; Duval et al. 2015) and efficient initiation is dependent on two gene features. First, the minimum requirement for translation initiation is that the start codon is accessible (Nakamoto 2006) and there is no stable secondary structure to embed the start codon (Scharff et al. 2011; Osterman et al. 2013). Second, presence of a Shine–Dalgarno (SD) sequence at the 5′ untranslated region (5′ UTR) upstream of start codon is prominent in prokaryotes (Nakagawa et al. 2017). The SD sequence facilitates recruitment of ribosome to start codon by pairing with the anti-SD sequence (Shine and Dalgarno 1974) located at the 3′ terminus of mature 16S rRNA (hereafter referred as 3′ tail).

Distinctions between SD-facilitated and SD-independent genes signal the importance of SD mechanism in bacterial translation initiation: genes with a well-positioned SD/anti-SD pair are more efficiently translated in bacteria (Schurr et al. 1993; Osterman et al. 2013; Abolbaghaei et al. 2017) and in their bacteriophages (Prabhakaran et al. 2015) than other genes; whereas translation in SD-independent genes are more reliant on reduced secondary structure stability at the initiation region (Scharff et al. 2011, 2017; Nakagawa et al. 2017) to compensate for the lack of SD sequence. Nonetheless, there have been many debates over the conservation and function of SD sequence in Cyanobacteria and in chloroplasts.

### Features of Functional SD Sequence in Bacteria

**Fig. 1.**
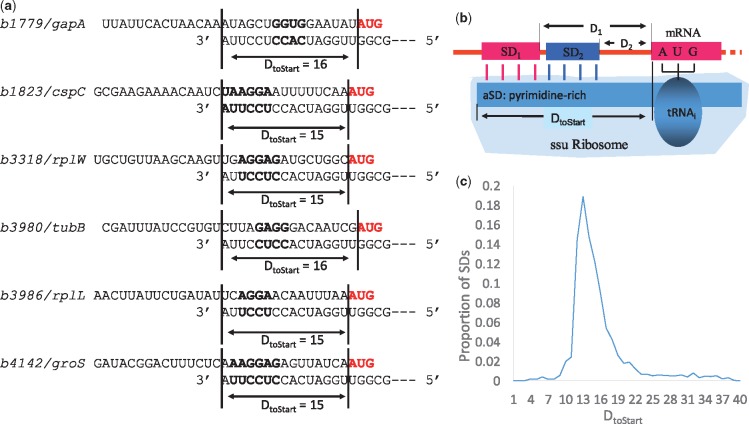
—A graphic model of SD/anti-SD interactions in *E. coli*, partially redrawn from Wei et al. (2017). (*a*) SD/anti-SD interactions in six genes whose proteins are abundantly produced in *E. coli*, showing that SDs (bold) can differ much from each other and in distance from start codon (red), but they have similar *D*_toStart_. (*b*) Illustration of *D*_toStart_ (number of nucleotides from the 3′ end of 16S rRNA to the nucleotide immediately upstream of the start codon), showing that SDs can be close to or far from the start codon but have the same *D*_toStart_. (*c*) *D*_toStart_ is strongly constrained within a narrow range for *E. coli* genes.

Good SD/anti-SD pairing that enhances translation initiation efficiency needs to meet two criteria (Xia 2018a). The first involves SD/anti-SD pairing position because this may serve as a major determinant for proper juxtaposition between start codon and ribosomal A site. An SD motif does not increase translation efficiency if it is not located at a proper distance away from the start codon (Hirose and Sugiura 1996; Hirose et al. 1998). Many researchers define a distance between the start or end of SD sequence and the start codon (Hirose et al. 1998; Ma et al. 2002; Starmer et al. 2006), but this is sometimes inadequate. For example, figure 1*a* lists six genes whose proteins are abundantly produced in *Escherichia coli*, together with their SD/anti-SD pairing. SD sequences can take different forms and bind to different anti-SD sequences on the 3′ tail. However, as illustrated in figure 1*b*, different SD sequences can be close to or far from the start codon but have the same distance from the 3′ tail to start codon. We recently proposed this improved measurement coined as *D*_toStart_ (Prabhakaran et al. 2015; Abolbaghaei et al. 2017; Wei et al. 2017) because the objective of SD/anti-SD pairing is to position the start codon at the ribosomal A-site to pair with the initiation tRNA. If *D*_toStart_ is important, then we expect it to be constrained within a narrow range, which is true in *E. coli* (fig. 1*c*) (Wei et al. 2017).

The second criterion involves SD/anti-SD binding strength. Studies often considered the anti-SD to consist of the canonical CCUCCU motif due to its high conservation (Nakagawa et al. 2017) and high binding affinity (Schurr et al. 1993). However, this focus on CCUCCU has excluded many other possible SD sequences. Recent studies suggest that SD/anti-SD pairing at this canonical motif may not be most preferred in bacteria (Wei et al. 2017), and intermediate SD/anti-SD complementarity at flanking sites improves bacterial gene expression (Hockenberry et al. 2017; Scharff et al. 2017; Wei et al. 2017). This is because strong SD/anti-SD pairing at CCUCCU may lead to ribosome stalling (Li et al. 2012; Zoschke and Bock 2018) that impedes the transition from initiation to elongation phase (Hockenberry et al. 2017); whereas intermediate complementarity may facilitate ribosome recruitment but does not inhibit ribosome movement along the mRNA.

### Controversies on SD Function in Cyanobacteria and Chloroplasts

Translation initiation is important to Cyanobacterial and chloroplast gene expression because there is no stable secondary structure to embed start codons in these species (Scharff et al. 2011). However, studies suggested that SD motifs are weakly conserved in Cyanobacteria and in plant chloroplasts that presumably originated from early endosymbiosis of ancient Cyanobacterium. First, translation initiation in some mRNAs relied on S1 protein in Cyanobacteria (Mutsuda and Sugiura 2006) and on an additional transacting factor in tobacco chloroplast (Plader and Sugiura 2003; Hirose and Sugiura 2004). This may explain why presence of SD sequence is reduced in both Cyanobacteria (Ma et al. 2002; Starmer et al. 2006; Nakagawa et al. 2010) and plastids (Scharff et al. 2017). Second, SD/anti-SD pairing distance was found to be weakly constrained in both *Synechocystis* sp. (Sazuka and Ohara 1996; Hirosawa et al. 1997; Ma et al. 2002) and tobacco chloroplast (Sugiura et al. 1998; Plader and Sugiura 2003). Nevertheless, these studies had focused on SD interactions with the canonical anti-SD motif CCUCCU and had measured pair distance from end of SD sequence to start codon, both are problematic as discussed above.

Consequently, there are conflicting views on the importance of SD-facilitated initiation in both Cyanobacteria and chloroplasts. Although a well-positioned SD sequence in plastids increased gene expression in some studies (Ye et al. 2001; Oey et al. 2009), mutating such SD sequences had no detectable effect on plastid initiation (Fargo et al. 1998; Nickelsen et al. 1999), and still others showed that weak SD/anti-SD complementarity increased plastid initiation (Scharff et al. 2017). In particular, the presence of SD sequence even reduced gene expression for one mRNA (Plader and Sugiura 2003). Hence, the importance of SD mechanism in chloroplast translation remains unclear, and this uncertainty raises skepticism over SD function in Cyanobacteria because chloroplasts presumably originated from Cyanobacteria.

If SD-facilitated initiation is unimportant in Cyanobacteria and chloroplasts, then the selection constraining SD usage and SD/anti-SD pairing most likely was lost or weakened prior to chloroplast endosymbiosis. On the contrary, if SD-facilitated initiation is important in Cyanobacteria but not important in chloroplasts, then SD usage and locational constraints would manifest in the former but not in the latter. This would also lead to the prediction that cyanophages, which depends on Cyanobacterial translation machinery for translation, should exhibit SD usage and SD/anti-SD pairing features similar to its Cyanobacterial hosts (and may resemble those in *E. coli* and *Bacillus subtilis*). This scenario would imply that SD function had been reduced in chloroplasts after having differentially evolved under host-symbiont coevolution away from Cyanobacteria.

### An Integrated Approach Sheds Light on Evolutionary Differences between Cyanobacteria and Chloroplast Translation Initiation

To test the hypotheses outlined above, we performed an integrated study using publicly available transcriptomic, genomic, and proteomic data of Cyanobacteria (*Microcystis aeruginosa* and *S.* sp.), chloroplasts (*Nicotiana tabacum* chloroplast and *Arabidopsis thaliana* chloroplast), and cyanophages (S-*SSM6a* and *S-SSM6b*) to quantify their differences in SD usage, locational constraints, and gene expression in presence and absence of SD sequence.

An issue that confounds the determination of functional SD sequences is the difficulty in reliably characterizing the 3′ terminus of mature 16S rRNA in bacteria (Lin et al. 2008; Wei et al. 2017) and in plastids (Gallaher et al. 2018). The annotated 3′ tails in *S.* sp., *M. aeruginosa* and chloroplasts were determined by automated computational predictions based on sequence homology against other bacterial species, and these predictions are often erroneous (Jones et al. 2007; Lin et al. 2008). These errors are problematic for two reasons: 1) determination of all potential SD sequences requires knowing the full extent of anti-SD sequence which constitutes the 3′ tail, and 2) identity of the 3′ tail is required to correctly measure SD/anti-SD locational constraints by *D*_toStart_.

The uncertainty in the 3′ tail results from the complexity in the 3′ maturation process of 16S rRNA. At least four RNases (RNase II, RNase R, PNPase, and YbeY) independently participates in this maturation process with unknown mechanisms (Sulthana and Deutscher 2013) and it is unclear whether they cleave the pre-16S rRNA 3′ end to the same length. Thus, the mature 16S rRNA may have a variety of end points as opposed to a single deterministic 3′ tail. Indeed, we have previously observed a degree of heterogeneity in the 3′ tail in five bacterial species (Silke et al. 2018).

Recently, we devised an RNA-Seq-based approach (Wei et al. 2017; Silke et al. 2018) to determine the most prominent 3′ tail in the rRNA pool, which led to the correction of 3′ tail mis-annotations in 12 bacterial species, including *S.* sp. To test the fidelity of our method, we have previously shown that the most prominent 3′ tail sequence determined by our RNA-Seq-based approach (Wei et al. 2017) matches the mature 16S rRNA 3′ terminus that was first experimentally validated by SD in *E. coli* and in *B. subtilis* (Shine and Dalgarno 1974, 1975). To our knowledge, similar experimental procedures have not been done in *S.* sp., *M. aeruginosa*, and chloroplasts. Hence, we applied our RNA-Seq-based approach to correct the potentially mis-annotated 3′ tail in these species.

Our results suggest differences between chloroplast and Cyanobacterial translation initiation. SD motif usage and *D*_toStart_ are strongly constrained in both Cyanobacteria and cyanophages, consistent with our prediction. In addition, SD-facilitated genes had significantly higher protein expression in comparison with other genes in both *S.* sp. and *M. aeruginosa*. Yet, in both chloroplasts *D*_toStart_ was loosely constrained and SD usage differed from those in Cyanobacteria. In addition, there was no significant difference in protein expression between SD-facilitated and other genes in chloroplasts. Furthermore, secondary structures at the 5′ UTR were significantly weaker in SD-independent genes to compensate for the lack of SD sequence in Cyanobacteria but not in chloroplasts. Although cyanophages mimic Cyanobacteria in SD usage because both have been under the same selection pressure for SD usage, chloroplasts lost this similarity likely because the need for SD-facilitated initiation was reduced in plastids during host-symbiont coevolution.

## Materials and Methods

### Processing Genomic, Proteomic, and RNA-Seq Data

The annotated genome of *S.* sp. PCC 6803 (NC_017277.1), *M.aeruginosa* NIES-843 (NC_010296.1), *N.tabacum* chloroplast (Z00044.2), *A.thaliana* chloroplast (NC_000932.1), and chloroplast genes from *A. thaliana* chromosomes 1–5: (NC_003070.8, 1.8, 4.8, 5.8, 6.8), cyanophages *S-SSM6a* (HQ317391.1), and *S-SSM6b* (HQ316603.1) were retrieved in GenBank format from the National Center for Biotechnology Information (NCBI) database (http://www.ncbi.nlm.nih.gov; last accessed October 23, 2019). *Synechocystis* sp. protein abundances (in ppm) from Wegener et al. (2010) and *M. aeruginosa* integrated protein abundances (in ppm) were retrieved from PaxDb 4.0 (Wang et al. 2015), and protein IDs were matched to NCBI gene IDs. *Nicotianatabacum* chloroplast protein abundances (in unique peptide counts) were retrieved from Wu and Yan (2018). *Arabidopsisthaliana* chloroplast protein abundances (in total spectral count) were retrieved from Ferro et al. (2010); here, we only considered proteins that were previously detected in the chloroplast (Zybailov et al. 2008).

High-throughput RNA sequencing data sets in *S.* sp. PCC6803 (PRJNA381210: SRX2694285, 6, 7, 8; PRJNA473849: SRX4145044, 5, 6), *M. aeruginosa* (strain KW, PRJNA421714: SRX3459379, 80; strain PCC 7806: PRJNA427104: SRX3501057, 8, 9), *N. tabacum* (PRJNA526208: SRX5495062, 75; PRJNA299013: SRX1342044, 5), and *A. thaliana* (PRJNA432917: SRX3647857, 58, 59, 60; PRJNA354600: SRX2367970) were retrieved from GEO Data sets in FASTQ format. All selected data sets are control runs for wild-type species, with the exception of *M. aeruginosa* KW (PRJNA421714), where S- and W-morphotype strains were employed. We chose RNA-Seq runs from plant species because there are no records of chloroplast-only RNA-Seq data sets in GEO Data sets, and because these experimental designs were tailored to study plastid transcripts (Pasoreck et al. 2016; Chang et al. 2017; Armarego-Marriott et al. 2019; Zhao et al. 2019).

These FASTQ data sets were then processed to eliminate low-quality reads and adapter sequences. We used Trimmomatic 0.38 (Bolger et al. 2014) to remove poor quality sequences with average Phred scores <20 (1% probability of base calling errors) (Ewing and Green 1998). Next, we used CutAdapt 1.17 (Martin 2011) to trim off flanking adapter sequences as listed in GEO Data set’s “*Construction protocol*” or in “*Publicatio*ns” (supplementary file S1, Supplementary Material online), with 10% error rate and retaining reads ≥25 nt to mitigate bias in expression levels (Williams et al. 2016). Then, processed FASTQ data sets were converted into FASTA+ format using ARSDA 1.1 (Xia 2017) (http://dambe.bio.uottawa.ca/Include/software.aspx; last accessed October 23, 2019). This step groups identical reads under a new unique ID indicating the number of identical copies (SeqID_No. of copies), reducing data size without loss of information.

### Characterizing the 3′ Tail Using RNA-Seq Data

To determine the 3′ tail, we mapped reads from each FASTA+ file onto the 16S rDNA query sequence. The FASTA+ files were converted into BLAST databases using the “*Create BLAST DB*” function in ARSDA. The first NCBI annotated 16S rRNA sequence was extracted from .gbk file in all species, and the BLAST query sequence was selected as the CCUCC core anti-SD motif plus 100 nt upstream and downstream (205 nt total query length). The query sequence was searched against the BLAST databases using BLAST (Altschul et al. 1990) implemented in DAMBE7 (Xia 2018b) with the following specifications: *E* value cutoff of 10^−10^, ungapped alignment, minimum match length of 25.

The most prominent 3′ tails were characterized based on map abundances that met two criteria: 1) they contained the core CCUCC and map within or close to 5′-GAUCACCUCCUU(U or A)-3′, and 2) they were the highest peaks with mapped counts appreciably greater than that at sites further downstream (greater than the combined counts of at-least five downstream bases). The basis for the first criterion was that CCUCC is conserved in 16S rRNA among bacterial species due to its presumed essential role in SD/anti-SD pairing, and that the genomic sequence 5′-GAUCACCUCCUU(U or A)-3′ is conserved across 277 prokaryote species (Nakagawa et al. 2010). The premise for the second criterion was that intermediate sequences between the mature and pre-16S rRNA are rapidly cleaved by RNases in *E. coli* (Cangelosi and Brabant 1997; Sulthana and Deutscher 2013), hence we expect to observe few map counts immediately downstream of the mature 3′ tail.

### Determining Functional SD Sequences Based on Pairing Potential and Location

The most prominent 3′ tails are 5′-GAUCACCUCCUUU-3′ in *S.* sp., 5′-GAUCACCUCCUU-3′ in *M. aeruginosa*, and 5′-GAUCACCUCC-3′ in both *N. tabacum* chloroplast and *A. thaliana* chloroplast. They were used as the complementary anti-SD sequence to identify ≥4 nt complementary SD sequences in non-pseudo genes. In addition, *S.* sp. and *M. aeruginosa* 3′ tails were each used to identify SD sequences in cyanophages. To identify putative SD sequences, we used DAMBE following the method used in previous studies (Prabhakaran et al. 2015; Abolbaghaei et al. 2017; Wei et al. 2017): 30 nt upstream of start codon of coding-DNA sequences were extracted and matched against the 16S rRNA 3′ tail with “*Analyzing 5*′*UTR*,” with minimum SD length = 4, maximum SD length = length of 3′ tail. DAMBE outputs the distance from the 16S rRNA 3′ end to the start codon denoted as *D*_toStart_. Notably, this 30 nt encompassed the −2 to −29 and −5 to −19 sites relative to start codon where most SD-like AGGAGG sequences were found in chloroplast (Hirose et al. 1998) and in *S.* sp. (Sazuka and Ohara 1996), respectively. The total number of putative SD sequences determined was 2,209 in *S.* sp., 3,860 in *M. aeruginosa*, 62 in *N. tabacum* chloroplast, and 50 in *A. thaliana* chloroplast.

In addition, DAMBE outputs the observed and expected site-specific anti-SD usages in the 3′ tail sequence (designated as *O_i_* and *E_i_*, respectively, hereafter, where *i* refers to site *i* in the 3′ tail sequence). If there is no selection constraint on anti-SD sequence, then *O_i_* is expected to be equal to *E_i_* (Wei et al. 2017). Therefore, an anti-SD site is preferred in pairing if its observed involvement was higher than that of expected (*O*:*E* >1). To test whether *O*:*E* >1 is significant, we applied the Central Limit Theorem for proportions. We defined the total sample size *n* as$\;\sum O_{i}$, the sum of *O_i_* at all *i* anti-SD sites; then the sample proportion of observed counts at each site was$\;p_{i}=O_{i}/n$, and the expected proportion at each site was $\hat{p_{i}}=\;E_{i}/n$. Because the sample size *n* was sufficiently large (in our case *n* = 8,709 in *S.* sp., 17,373 in *M. aeruginosa*, 283 in *N. tabacum* chloroplast, and 314 in *A. thaliana* chloroplast), by CTL $p_{i}\;$approximates a normal distribution $N(\hat{p_{i}},\;\sqrt{\hat{p_{i}}(1-\hat{p_{i}})/n}$) and the Z-score $Z_{i}=\;p_{i}\;-\;\hat{p_{i}}/\sqrt{\hat{p_{i}}(1-\;\hat{p_{i}})/n}$. Because we were only interested in testing whether *O*:*E* >1 is significant, we took an upper-tailed hypothesis test with $H_{1}:\;p_{i}>\;\hat{p_{i}}$ and $H_{0}:\;p_{i}=\;\hat{p_{i}}$, and rejected the null hypothesis with *P* < 0.05 if $Z_{i}\;\geq 1.645$.

To examine SD role in gene expression, we defined genes with nonzero protein abundances as SD-facilitated if the determined putative SD sequences were located within the preferred *D*_toStart_ ranges of 11–21 nt in *S.* sp., 11–20 nt in *M. aeruginosa*, 8–16 nt in *S-SSM6a*, 10–21 nt in *S-SSM6b*, 8–16 nt in *N. tabacum* chloroplast, and 10–21 nt in *A. thaliana* chloroplast. These optimal ranges were determined because they encompassed the confined *D*_toStart_ peaks in Cyanobacteria and cyanophages and constituted the most abundantly mapped region in chloroplasts (illustrated in fig. 3, % of SDs they encompass described in supplementary file S2, Supplementary Material online). Whereas all other genes with nonzero protein abundances were defined as SD-independent genes.

### Measuring Translation Efficiency with Protein per Transcript

To measure protein per transcript, mRNA abundance was profiled in ARSDA. A query file containing coding DNA sequences of all SD-facilitated and SD-independent genes were built in FASTA format. This query sequence was searched against the BLAST databases using ARSDA’s “*Gene expression from BLAST Database*,” with the following specifications: *E* value = 10^−10^, ungapped alignment, minimum match length = 25, Max target hits = 1,000,000. ARSDA outputs the normalized map abundances in Fragments per kilobase of transcript per million mapped reads (FPKM). For each gene, the averaged FPKM values were calculated from FPKM values obtained in all selected RNA-Seq replicates within the same BioProject. Finally, protein per transcript was calculated as$\;\frac{\mathrm{protein}\;\mathrm{abundance}}{\mathrm{average}\;\mathrm{mRNA}\;\mathrm{FPKM}}$.

### Determining Secondary Structure Stability at the Translation Initiation Region

In SD-facilitated and SD-independent genes, we measured folding energy of a 40 nt sequence at the 5′ UTR following the approach by Tuller et al. (2010) and Kudla et al. (2009). In particular, Tuller et al. (2010) measured the folding energy of a 40 nt sequence immediately upstream of the start codon. We designated this sequence as the translation initiation region and followed this approach, because in our study for SD role in Cyanobacteria and chloroplasts our objective was to compare the accessibility of the upstream region having or lacking a SD sequence. To measure folding energy, we used minimum folding energy (MFE, kcal/mol) via Vienna RNA Folding Library (Hofacker 2003) implemented in DAMBE, with options: no lonely pair, Window size = 40, StepSize = 1. Importantly, this 40 nt encompassed all putative SD sequences determined herein.

## Results

### The 3′ Tail Is Required to Determine Preferred Anti-SD Motifs

**Fig. 2.**
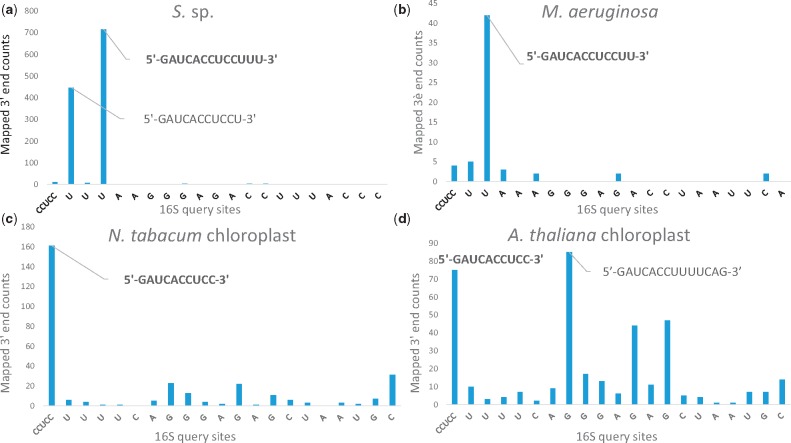
—Site-specific counts of RNA-Seq read 3′ ends mapped to the 16S rDNA query sequence in (*a*) *S.* sp., (*b*) *M. aeruginosa*, (*c*) *N. tabacum* chloroplast, and (*d*) *A. thaliana* chloroplast. The first site query is the core CCUCC motif, followed by 20 downstream sites. The identity of the most prominent 3′ tail that meets the two identification criteria is highlighted in bold.

We characterized the most prominent 3′ tail in *S.* sp. (5′-GAUCACCUCCUUU-3′), *M. aeruginosa* (5′-GAUCACCUCCUU-3′), *N. tabacum* chloroplast (5′-GAUCACCUCC-3′), and *A. thaliana* chloroplast (5′-GAUCACCUCC-3′) (fig. 2; see Materials and Methods section for criteria used in 3′ tail determination) and corrected the mis-annotated *S.* sp. 5′-GAUCACCUCCUUUAAGGG-3′ (NC_017277.1), *M. aeruginosa* 5′-GAUCACCUCCUUA-3′ (NC_010296.1), *N. tabacum* chloroplast 5′-GAUCACCUCCUUU-3′ (Z00044.2), and *A. thaliana* chloroplast 5′-GAUCACCUCCUUU-3′ (NC_000932.1). Indeed, automated annotations of 16S rRNA are often erroneous (Starmer et al. 2006; Jones et al. 2007; Lagesen et al. 2007; Lin et al. 2008). Notably, a secondary 3′ tail peak was observed from both independent RNA-Seq studies in *S.* sp. (5′-GAUCACCUCCU-3′) and in *A. thaliana* chloroplasts (5′-GAUCACCUCCUUUUCAG-3′). Similarly, we have previously reported a degree of heterogeneity in the 3′ tail in four other bacterial species (Silke et al. 2018). Together, they suggest that the mature 16S rRNA molecule may have a variety of end points in some species.

Next, we showed that terminal bases in the most prominent 3′ tail are just as important in Cyanobacteria as they are in *E. coli* or *B. subtilis* because they are often more preferred in SD/anti-SD pairing than the canonical CCUCC (or CCUCCU) motif (Wei et al. 2017). We defined an anti-SD site to be preferred if the number of times the site was observed in pairing with putative SD sequence was significantly greater than expected (*O*:*E* >1, *P* < 0.05, upper-tailed; see Materials and Methods section). Here, we designated the characterized 3′ tails (fig. 2) as the anti-SD sequences. Table 1 shows that the preferred anti-SD sequence is 5′-UCUCCUUU-3′ in *S*. sp., 5′-UCCUU-3′ in *M. aeruginosa*. However, a preferred anti-SD sequence with ≥4 nt could not be obtained from *N. tabacum* chloroplast or *A. thaliana* chloroplast.

**Table 1:** Anti-SD Site-Specific Pairing Preference for Putative SD Sequences in Cyanobacterial and Chloroplast Non-Pseudo Genes

Anti-SD Site	*O*:*E S*. sp.	*O*:*E M. aeruginosa*	*O*:*E N. tabacum* Chloroplast	*O*:*E A. thaliana* Chloroplast
G	0.97	1.03	0.71	1.45
A	0.98	1.13	1.22	1.32
U	0.79	0.92	0.88	1.54
C	0.69	0.79	0.74	1.27
A	0.72	0.78	0.76	1.05
C	0.68	0.65	0.69	0.85
C	0.94	0.87	0.97	0.59
U	1.11	1.11	1.19	0.67
C	1.15	1.21	1.73	0.77
C	1.35	1.30	2.75	0.73
U	1.36	1.58		
U	1.33	1.54		
U	1.63			

Note.—*O*:*E* denotes the ratio of observed to expected pairs. In red is anti-SD sites with significant *O*:*E* >1 (*P* < 0.05, upper-tailed test; the specific *Z*-scores are provided in supplementary file S2, Supplementary Material online).

### SD Usage and SD/Anti-SD Locational Constraints Are Similar between Cyanobacteria and Cyanophages but Differ in Chloroplasts

**Fig. 3.**
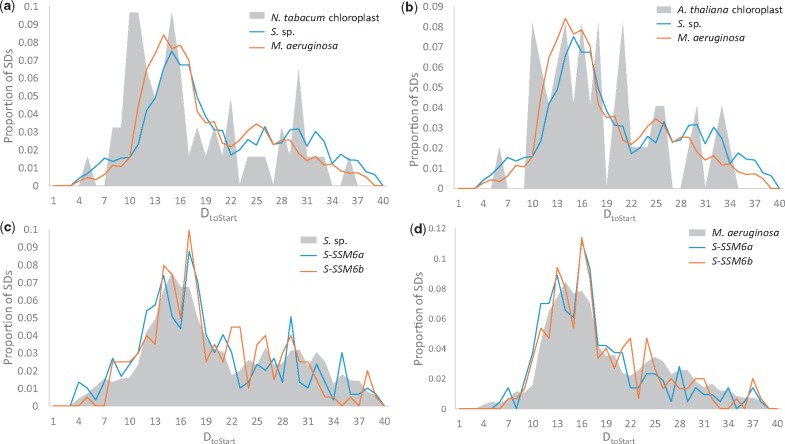
—Comparisons in *D*_toStart_ among Cyanobacteria, cyanophages, and chloroplasts. *D*_toStart_ constraints differ between Cyanobacteria (*S.* sp. and *M. aeruginosa*) and (*a*) *N. tabacum* chloroplast and (*b*) *A. thaliana* chloroplast. Whereas *D*_toStart_ constraints in (*c*) *S.* sp. and in (*d*) *M. aeruginosa* are well mimicked by cyanophages (*S-SSM6a* and *S-SSM6b*). Phage *D*_toStart_ in (*c*) was obtained using *S.* sp. anti-SD 5′-GAUCACCUCCUUU-3′, and in (*d*) was obtained using *M. aeruginosa* anti-SD 5′-GAUCACCUCCUU-3′. Shaded gray are *D*_toStart_ constraints for outgroup species subjected to comparison.

**Fig. 4.**
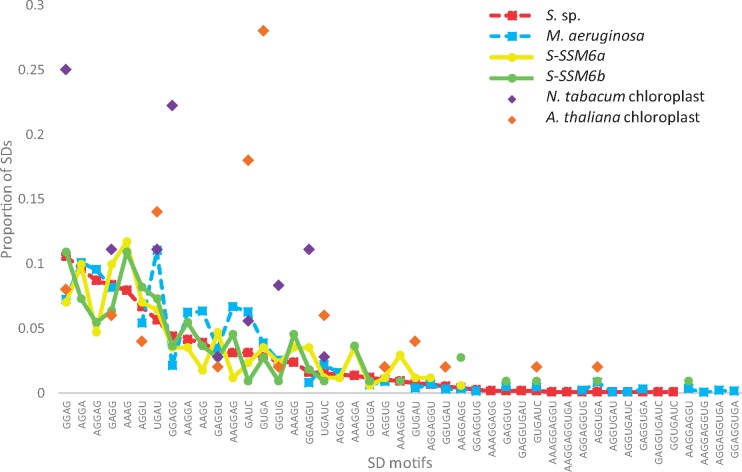
—Comparisons in SD motif usage among Cyanobacteria, cyanophages, and chloroplasts. Usage of SD motifs at optimal *D*_toStart_ is comparable among Cyanobacteria *S.* sp. (red), *M. aeruginosa* (blue), cyanophage *S-SSM6a* (yellow), and cyanophage *S-SSM6b* (green), but varies appreciably in *N. tabacum* chloroplast (purple) and in *A. thaliana* chloroplast (orange). All data points presented have nonzero SD proportions.

SD locational constraints and motif usage differ between Cyanobacteria and chloroplasts. Figure 3*a* and *b* highlight that *D*_toStart_ is similarly confined in *S.* sp. and *M. aeruginosa* but differ between Cyanobacteria and chloroplasts. In contrast, figure 3*c* and *d* shows that *D*_toStart_ constraints in Cyanobacteria are well mimicked by cyanophages *S-SSM6a* and *S-SSM6b*. In addition, we found that usage of SD motifs located at optimal *D*_toStart_ (fig. 4; see Materials and Methods section for optimal *D*_toStart_ identification) were similar between *S.* sp. and *M. aeruginosa* and they were well mimicked by cyanophages; however, SD motif usage varied drastically in chloroplasts. A test for ordinal association using Kendall’s tau-*b* statistic with adjustment for tied ranks showed SD motif usage is highly correlated between *S.* sp. and *M. aeruginosa* (*τ_b_* = 0.606, *P* < 0.00001, two-tailed) and cyanophages (*S-SSM6a*: *τ_b_* = 0.812, *P* < 0.00001, two-tailed; *S-SSM6b*: *τ_b_* = 0.809, *P* < 0.00001, two-tailed) but only moderately correlated between *S.* sp. and chloroplasts (*N. tabacum* chloroplast: *τ_b_* = 423, *P* = 0.00057, two-tailed; *A. thaliana* chloroplast: *τ_b_* = 0.339, *P* = 0.00482, two-tailed). In both figures 3 and 4, we considered all putative SD sequences (2,209 in *S.* sp., 3,860 in *M. aeruginosa*, 62 in *N. tabacum* chloroplast, 50 in *A. thaliana* chloroplast, 297 in *S-SSM6a*, and 201 in *S-SSM6b*).

Figure 2 illustrates a secondary 3′ tail peak in *S.* sp. (5′-GAUCACCUCCU-3′) and in *A. thaliana* chloroplast (5′-GAUCACCUCCUUUUCAG-3′). We have additionally determined putative SD sequences using these alterative 3′ tails and analyzed *D*_toStart_ constraints and SD motif usage at optimal *D*_toStart_ (supplementary fig. S1, Supplementary Material online). Similarly, supplementary figure S1*a* and *b*, Supplementary Material online highlight that *D*_toStart_ constraints resemble among Cyanobacteria and cyanophages, whereas chloroplast SD sequences lost this resemblance. In addition, supplementary figure S1*c*, Supplementary Material online shows that SD motif usage is again comparable between *S.* sp. and *M. aeruginosa* (*τ_b_* = 0.621, *P* < 0.00001, two-tailed) even though AAGGAG, AAGG, and AAGGA could not be determined in *S.* sp. when the shorter anti-SD 5′-GAUCACCUCCU-3′ was employed; whereas similarities in SD motif usage are again weaker between Cyanobacteria and chloroplasts (*N. tabacum* chloroplast: *τ_b_* = 0.580, *P* < 0.00001, two-tailed; *A. thaliana* chloroplast: *τ_b_* = 0.169, *P* = 0.132, two-tailed).

### SD-Facilitated Initiation Increases Cyanobacterial but Weakly Influences Chloroplast Gene Expression

**Fig. 5.**
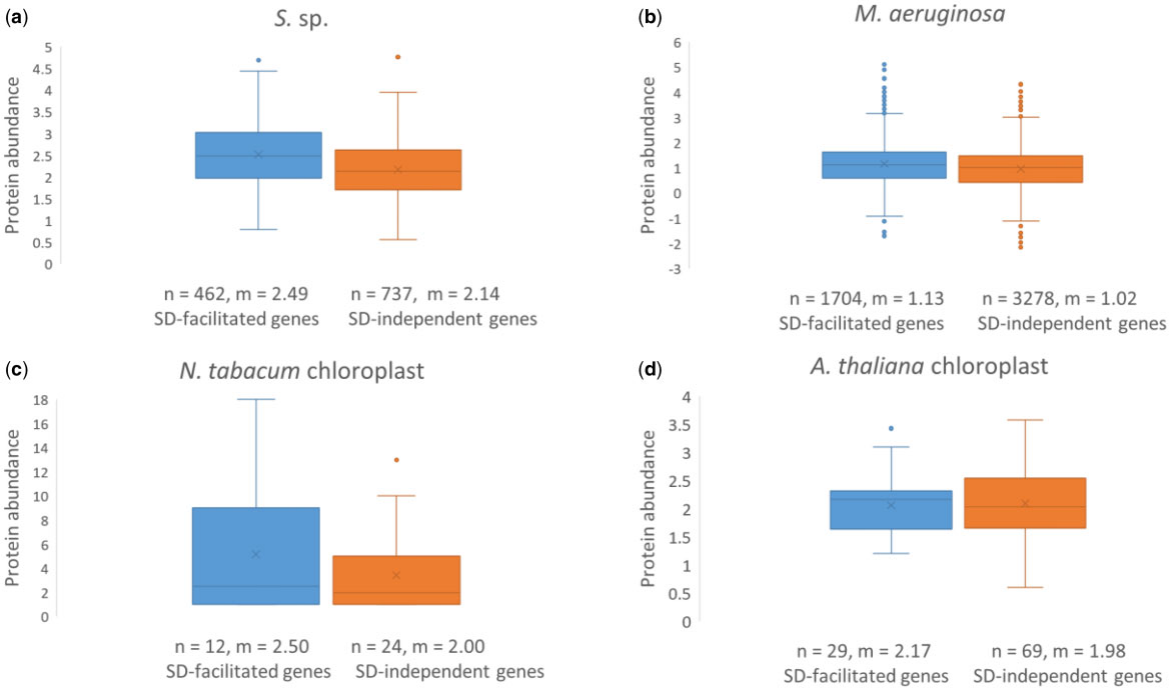
—Contrasts in protein abundances between SD-facilitated genes (SD/anti-SD pair ≥4 nt and SD within optimal *D*_toStart_) and SD-independent genes. Protein abundances in Cyanobacteria (*a*, *b*) were measured by log(ppm) and retrieved from PaxDb database, in *N. tabacum* chloroplast (*c*) were measured by number of unique peptides and retrieved from Wu and Yan (2018), and in *A. thaliana* chloroplast (*d*) were measured by log(total spectral counts) and retrieved from Ferro et al. (2010) curated as plastid proteins by Zybailov et al. (2008). All genes are non-pseudo with protein abundance >0. For each gene set, the number of genes (*n*) and median (*m*) is denoted under the boxplots.

Results above imply SD-facilitated initiation is crucial in Cyanobacteria but not in chloroplasts. To estimate SD role in gene expression, we measured the impact a well-positioned SD sequence (with optimal *D*_toStart_) has on Cyanobacterial and chloroplast protein abundance. We observed a significant increase in protein abundance for SD-facilitated genes over other genes in *S.* sp. (fig. 5*a*, Wilcoxon rank sum test with continuity correction: *P* < 0.00001) and in *M. aeruginosa* (fig. 5*b*, Wilcoxon rank sum test with continuity correction: *P* < 0.00001), but the contrast was not significant in *N. tabacum* chloroplast (fig. 5*c*, Wilcoxon rank sum test with continuity correction: *P* = 0.429) or in *A. thaliana* chloroplast (fig. 5*d*, Wilcoxon rank sum test with continuity correction: *P* = 0.460). Indeed, a well-positioned SD/anti-SD pair improves protein expression in bacteria (Ma et al. 2002; Osterman et al. 2013) but may influence only a few mRNAs in chloroplasts (Hirose et al. 1998; Scharff et al. 2017).

To contrast translation efficiency between SD-facilitated and SD-independent genes, we additionally calculated protein per transcript (protein abundance/average mRNA FPKM; supplementary fig. S2, Supplementary Material online) which normalized protein abundance to mRNA transcript abundance. For every species, we had retrieved RNA-Seq data sets from two independent studies; therefore, two protein per transcript values were calculated for each gene (see Materials and Methods section). Supplementary figure S2, Supplementary Material online shows that protein per transcript values are significantly higher in SD-facilitated genes than SD-independent genes in *S.* sp. (Wilcoxon rank sum test with continuity correction: *P* < 0.0001 in both panels), but protein per transcript values do not differ significantly between the two gene sets in *M. aeruginosa* (Wilcoxon rank sum test with continuity correction: *P* = 0.201, *P* = 0.132), *N. tabacum* chloroplast (Wilcoxon rank sum test with continuity correction: *P* = 0.931, *P* = 0.900), or *A. thaliana* chloroplast (Wilcoxon rank sum test with continuity correction: *P* = 0.210, *P* = 0.135). Hence, SD-mediated translation initiation significantly increases mRNA translation efficiency in at least *S.* sp. but not in chloroplasts.

### Translation Initiation Region in SD-Independent Genes Is Compensated with Weaker Secondary Structure Stability in Cyanobacteria but Not in Chloroplasts

**Fig. 6.**
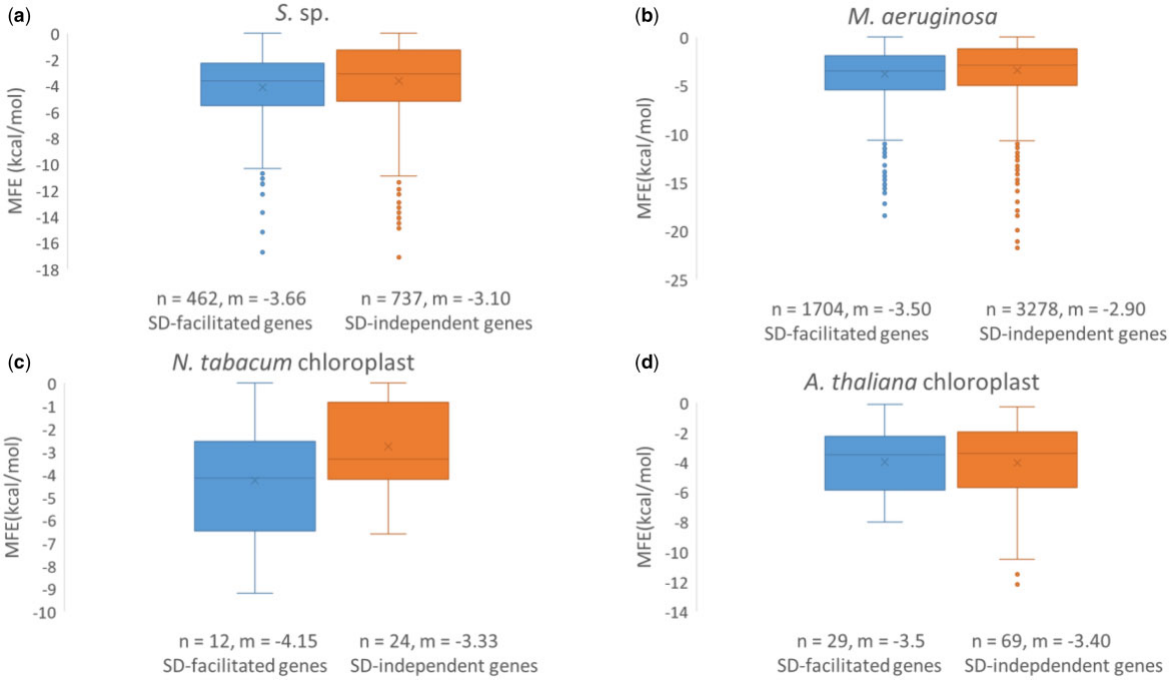
—Contrasts in MFE (kcal/mol) at the 40 nt initiation region immediately upstream of the start codon between SD-facilitated genes (SD/anti-SD pair ≥4 nt and optimal *D*_toStart_) and SD-independent genes in (*a*) *S.* sp., (*b*) *M. aeruginosa*, (*c*) *N. tabacum* chloroplast, and (*d*) *A. thaliana* chloroplast. All genes are non-pseudo with nonzero protein abundances. For each gene set, the number of genes (*n*) and median (*m*) is denoted under the boxplots.

In figure 6 we compared the accessibility of ribosome binding sites between SD-facilitated and SD-independent genes in Cyanobacteria and chloroplasts. We defined this translation initiation region to consist of 40 nt immediately upstream of the start codon because it encompassed all putative SD sequences in SD-facilitated genes. We observed a significant decrease in secondary structure stability (smaller—kcal/mol) in SD-independent genes over SD-facilitated genes in *S.* sp. (Wilcoxon rank sum test with continuity correction: *P* = 0.00018) and in *M. aeruginosa* (Wilcoxon rank sum test with continuity correction: *P* < 0.00001), but not in *N. tabacum* chloroplast (Wilcoxon rank sum test with continuity correction: *P* = 0.158) or in *A. thaliana* chloroplast (Wilcoxon rank sum test with continuity correction: *P* = 0.840). This result is consistent with an previous study (Scharff et al. 2011) that showed weaker secondary structure stability at the 5′ UTR in genes lacking a SD sequence in Proteobacteria and in Cyanobacteria.

**Fig. 7.**
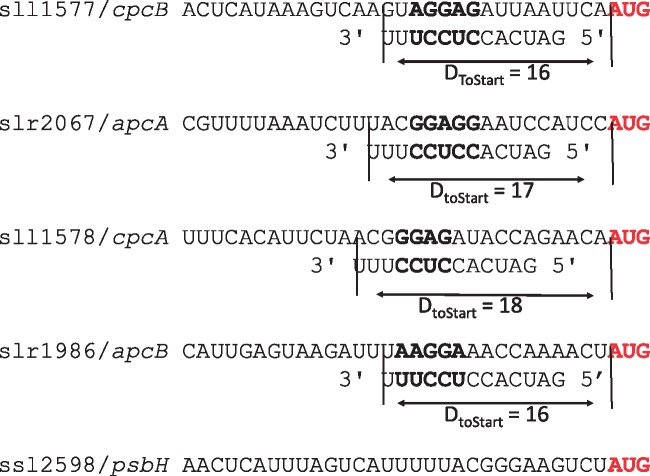
—SD/anti-SD interactions can be found between mRNA and 3′ tail of 16S rRNA in four of the five *S.* sp. genes with the most abundant proteins, but not in ssl2598/*psbH*. In red is the start codon, in bold the SD/anti-SD pair.

Figure 7 illustrates an example of the evolutionary advantage for SD-independent genes to have weakened secondary structure stability at translation initiation region in *S*. sp. Among the five genes with most abundant proteins in *S.* sp. characterized by Wegener et al. (2010), four genes exhibit properly positioned SD/anti-SD base-pairing interactions, but one ssl2598/*psbH*, whose protein happens to be the most abundant of all genes, cannot form SD/anti-SD interactions with a *D*_toStart_ near the peak shown in figure 3*a*. One would predict that ssl2598/*psbH* would need to have reduced secondary structure stability near the translation initiation region to compensate for the lack of a well-positioned SD/anti-SD. We calculated MFE for the 40 nt immediately upstream of the start codon of these five genes. The result is consistent with the expected compensation effect between SD/anti-SD and secondary structure stability. The MFE (kcal/mol) is −10.95 for sll1577/*cpcB*, −5.81 for slr2067/*apcA*, −7.78 for sll1578/*cpcA*, and −7.08 for slr1986/*apcB*, but only −2.51 for ssl2598/*psbH*, with more negative MFEs corresponding to stronger secondary structures.

## Discussion

There have been many debates over the conservation and function of SD sequence in Cyanobacteria and in chloroplasts. Elucidating SD role in Cyanobacterial and in plastid translation initiation is crucial to understand how these species may have differentially evolved. One of the first indications of differences between bacteria and chloroplast translation came from isolation of chloroplast ribosomal proteins that have no homologs in *E. coli* (Plader and Sugiura 2003). Because chloroplasts are presumably derived from Cyanobacteria through endosymbiosis, this difference between chloroplast and *E. coli* may be traced in Cyanobacteria. Therefore, contrasts in SD usage and function between chloroplasts and Cyanobacteria have two distinct implications. If the location of SD sequence is poorly constrained and SD sequences weakly influence gene expression in both Cyanobacteria chloroplasts, then SD role in translation initiation may have already been weakened in Cyanobacteria prior to chloroplast endosymbiosis. However, if features of functional SD sequence in *E. coli* are retained in Cyanobacteria and are mimicked by cyanophages, then the SD mechanism is crucial for Cyanobacterial translation initiation and chloroplast SD function may have been weakened as a result of host-symbiont coevolution after symbiosis. However, because the most recent common ancestor (MRCA) of chloroplasts is unknown, and because the cyanobacterial species we studied may not be representative of MRCA, our finding does not exclude the possibility that the importance of SD/anti-SD base-pairing might have already been decreased in MRCA.

Knowing the full extent of 3′ tail is required to identify the complete pool of functional SD motifs. We had previously devised an RNA-Seq-based approach to correct the mis-annotated 3′ tail in several bacterial species (Wei et al. 2017; Silke et al. 2018). Here, we rectified the mis-annotated 3′ tails in *S.* sp., *M. aeruginosa*, *N. tabacum* chloroplast, and *A. thaliana* chloroplast. Using an *O*:*E* metric we showed that the preferred anti-SD motif is 5′-UCUCCUUU-3′ in *S*. sp. and 5′-UCCUU-3′ in *M. aeruginosa*, but a preferred anti-SD motif could not be determined in chloroplasts (table 1). Like in *E. coli* and in *B. subtilis*, the 3′ terminal sequences extending past the canonical CCUCCU are preferred in SD/anti-SD pairing in Cyanobacteria. This finding corroborates recent studies that suggested intermediate SD/anti-SD complementarity increases bacterial gene expression (Hockenberry et al. 2017; Scharff et al. 2017); whereas strong complementarity at CCUCCU may lead to ribosome stalling in *E. coli* (Li et al. 2012). In addition, SD/anti-SD pairing position is crucial to SD function, and characterizing the most prominent 3′ tail allowed us to accurately determine SD/anti-SD locational constraints using *D*_toStart_ and to find functional SD sequences having proper juxtapositioning between the start codon and ribosomal A site.

Our results provided three lines of evidence to suggest that the SD mechanism is important for Cyanobacterial translation initiation. First, we showed that SD/anti-SD pairing location is well constrained as measured by *D*_toStart_ in Cyanobacteria (fig. 3*a*). This constraint was similarly found for SD usage in *E. coli* and *B. subtilis* (Wei et al. 2017). Second, both SD/anti-SD locational constraints and SD motif usage in Cyanobacteria were well mimicked by cyanophages (figs. 3*c*, 3*d*, and 4*a*). These similarities indicate host-phage specificity and emphasizes the importance of SD-facilitated initiation in Cyanobacteria because the same patterns were also observed in *E. coli* and coliphages (Prabhakaran et al. 2015). Third, SD-facilitated genes had higher protein abundance whereas SD-independent genes compensated by having weaker secondary structure stability at the initiation region (figs. 5 and 7). Because recruitment of ribosome is slower in absence of SD sequence, it is evolutionarily more advantageous for SD-independent genes in bacteria to adopt a structure-less initiation region to facilitate ribosome loading (Nakamoto 2006; Scharff et al. 2011). Thus, SD sequence plays an important role in Cyanobacterial gene expression.

In contrast, there were notable differences in SD usage between chloroplasts and Cyanobacteria (fig. 4). In addition, chloroplast *D*_toStart_ was weakly constrained in comparison with Cyanobacteria (fig. 3). These imply weakened SD function in chloroplasts in support for the theory that the mechanisms of translation initiation are subjected to different evolutionary pressure between bacteria and symbiont. Resultantly, chloroplast SD-facilitated genes did not have significant increase in protein expression (fig. 5), and other genes did not compensate by having weaker secondary structure at the 5′ UTR (fig. 6). A caveat of this study is the small number of unique proteins retrievable from chloroplasts: only 38 in *N. tabacum* chloroplast from Wu and Yan (2018) and 98 in *A.* thaliana chloroplast from Ferro et al. (2010) that were previously detected as plastid proteins by Zybailov et al. (2008). This may be attributed to the fact that chloroplast genomes have shrank significantly due to host-endosymbiont coevolution (Timmis et al. 2004) and about 120 genes remain in present-day green plant chloroplasts (Li et al. 2012; Zoschke and Bock 2018). This entails that SD sequence in remaining genes would resemble optimal ones used in Cyanobacteria should the SD mechanism be crucial to chloroplast translation initiation; however, this was not the case.

To examine SD role in translation efficiency, we have additionally contrasted protein per transcript values between SD-facilitated genes and SD-independent genes. Similar to figure 5, we found that protein per transcript levels were significantly higher in SD-facilitated genes than SD-independent genes in *S.* sp. but not in chloroplasts (supplementary fig. S2, Supplementary Material online). Yet, we observed no significant difference in protein per transcript between the two gene sets in *M. aeruginosa*. A plausible explanation for this result is that *M. aeruginosa* gene coverage in mRNA profiling is poor (88.8% coverage from PRJNA421714 and 76.4% coverage from PRJNA427104 for 4,982 genes of interest, as opposed to >95% coverage for all other species). Similarly, in PRJNA427104, the authors’ provided FPKM file has about 47% gene coverage. A plausible explanation for this poor gene coverage is that annotated genes in *M. aeruginosa* (NC_010286.1) are putative and determined by automated predictions based on sequence homology against other cyanobacterial species (Kaneko et al. 2007). A degree of mis-annotations is to be expected from such automated predictions (Schnoes et al. 2009). Nevertheless, supplementary figure S2, Supplementary Material online shows that SD-facilitated initiation modestly increases translation efficiency in the Cyanobacteria *S.* sp. but not in chloroplasts.

An alternative 3′ tail was found in *S.* sp. (5′-GAUCACCUCCU-3′) and *A. thaliana* chloroplast (5′-GAUCACCUCCUUUUCAG-3′). Similarly, we have previously reported 3′ tail heterogeneity in four other bacterial species (Silke et al. 2018). These suggest that in some species the mature 16S rRNA molecules may have a variety of end points, rather than a single deterministic 3′ end, because the pre-16S rRNA 3′ end is independently processed by five different RNases (Sulthana and Deutscher 2013). Alternatively, a secondary peak extending far downstream from the conserved genomic sequence 5′-GAUCACCUCCUU(U or A) (Nakagawa et al. 2010) may constitute the pre-16S rRNA, which is accumulated (Sulthana and Deutscher 2013) because the localization of RNases to this precursor sequence is a rate limiting step during 3′ maturation. Nonetheless, we determined putative SD sequences using this alternative 3′ tail in *S.* sp. and *A. thaliana* chloroplast and investigated *D*_toStart_ constraints and SD motif usage (supplementary fig. S1, Supplementary Material online). Again, we found that both *D*_toStart_ and SD motif usage were similarly between *S.* sp. and *M. aeruginosa*, but they differed appreciably in *A. thaliana* chloroplast.

Our results suggest that the SD mechanism is crucial to Cyanobacterial gene expression, and cyanophages mimic Cyanobacteria in SD sequence usage because it is evolutionarily advantageous for bacteriophages to take up features that increase host gene expression. On the contrary, chloroplasts lost this similarity because the need for SD-facilitated initiation is reduced in plastids having much reduced genome size (Timmis et al. 2004), different ribosomal proteins (Plader and Sugiura 2003), and other possible mechanisms of initiation (Plader and Sugiura 2003; Hirose and Sugiura 2004) as a result of host-symbiont coevolution.

## Supplementary Material

Supplementary data are available at *Genome Biology and Evolution* online.

## Supplementary Material

evz227_Supplementary_Data
